# Supplementary material for: Design and evaluation of a problem-based learning VR module for apparel fit correction training
Source: PLoS One. 2025 Jan 9;20(1):e0311587. doi: 10.1371/journal.pone.0311587 (PMC11717263; doi:10.1371/journal.pone.0311587)
Supplement: S1 Fig — (PDF) [file pone.0311587.s001.pdf]

Supporting information

S1 Fig. Iterative Fit Correction Tasks. (Source: Authors' own work)

I. Square neck top with bust darts		
**Fit issue (F)**	**Reason**	**Solution (S)**
F1. The back hem dropped lower, the front hem flared, and the side seam swung toward the front	The horizontal base line was not parallel to the ground at the back	Lift back panel at the shoulder
F2. Draglines radiating from the side seam	Excessive suppression of the side seam at the waist	Reduce the curvature of the side seam
F3. Discomfort at armhole	Insufficient armhole depth	Increase armhole depth
F4. Gapping at the armhole and dipping under the bust	Inadequate 3D shaping of the bust	Increase bust dart in-take

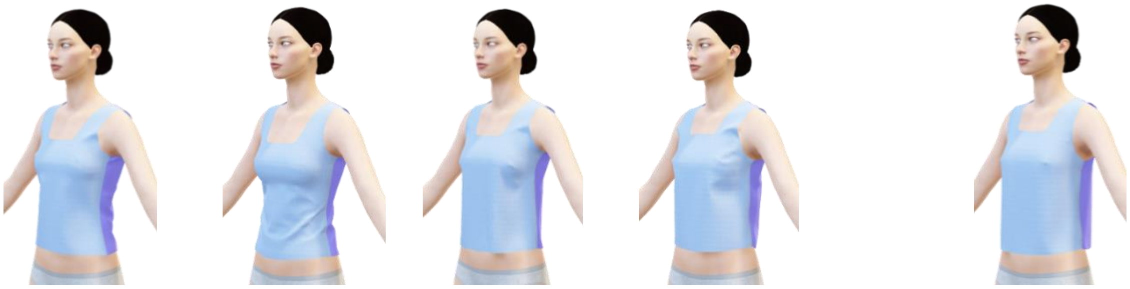

F1 F2 F3 F4 S

II. Pants with waist darts		
**Fit issue (F)**	**Reason**	**Solution (S)**
F1. Drag lines from the front thigh to the side seam	Pants were tight at the buttocks and side seam	Add extra curvature to the side seam to accommodate the contours of the body
F2. Bubble forming at the front rise	Excess front rise length	Making the rise curve deeper and removing excess from the waist
F3. Waistband dipping at the center back	Lack of back rise	Increase the back rise length

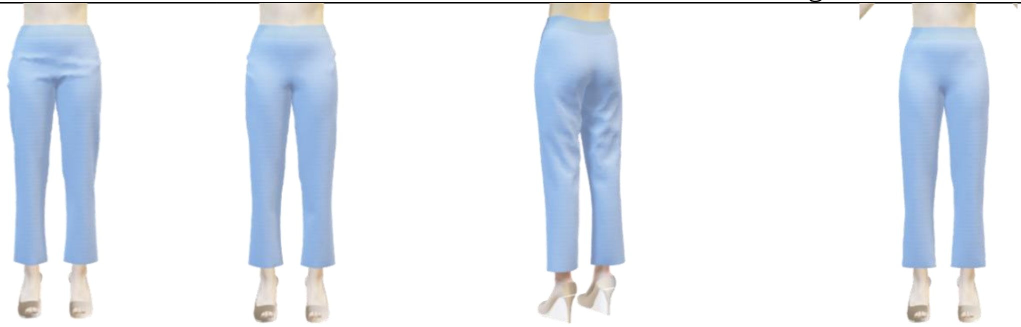

F1 F2 F3 S

S1 Fig. Iterative Fit Correction Tasks (continued).

III. Full-sleeve raglan t-shirt		
**Fit issue (F)**	**Reason**	**Solution (S)**
F1. Folds around the front shoulder area	The shirt was loose overall, and the excess fabric created folds	Eliminate excess fabric from the front panel at the front panel – raglan sleeve seam
F2. The overall garment was loose	Excess circumference measurements	Remove fabric from side seams
F3. Draglines at the upper back	Shortage of length in the back panel	Increase height along the sleeve seam
F4. Draglines around the sleeve and shoulder	Armhole circumference measurement too large	Reduce armhole circumference

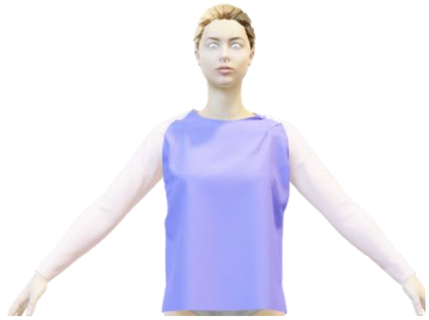

F1

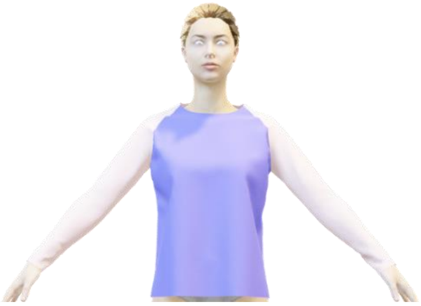

F2

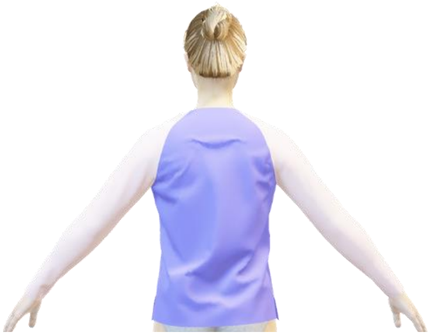

F3

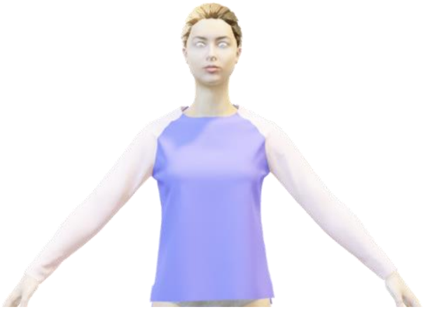

F4

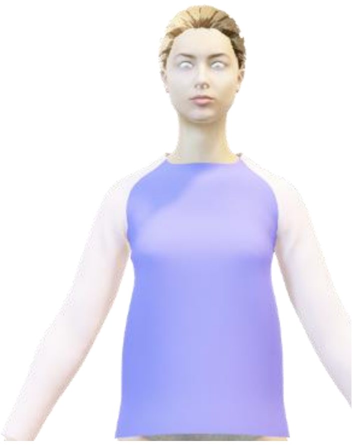

S

S1 Fig. Iterative Fit Correction Tasks (continued).

IV. Sleeveless dress with princess seam		
**Fit issue (F)**	**Reason**	**Solution (S)**
F1. Horizontal folds at the lower back	The hemline of the upper back panel not being parallel to the ground	Straighten the back waistline
F2. Folds radiating from the bust to the armhole	Excess fabric at the armhole	Increase bust dart intake
F3. Drag lines originating from the bust	Tightness at the bust because of lack of 3D shaping	Add extra fabric at the princess seam

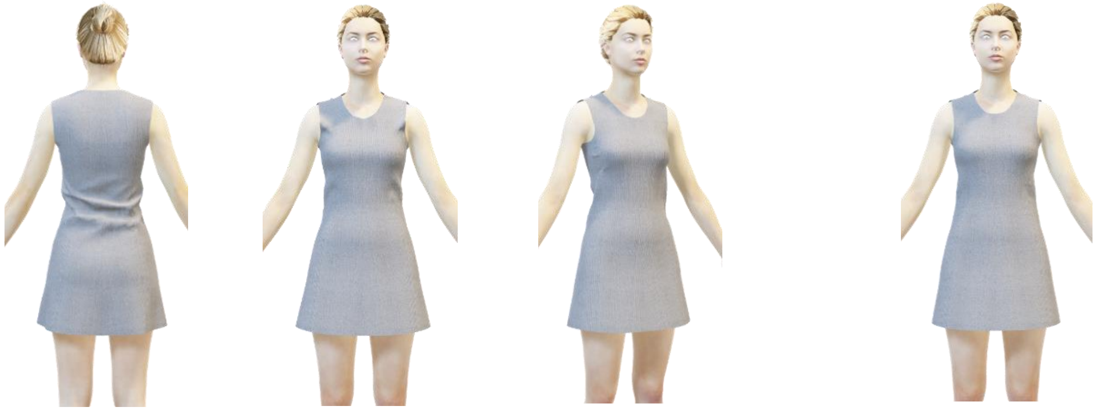

F1 F2 F3 S

V. A-line Skirt		
**Fit issue (F)**	**Reason**	**Solution (S)**
F1. Side seam pull to the back	S-shaped figure	Lower center front waist
F2. Horizontal folds at the back panel above the buttocks	Horizontal base line not parallel to the ground	Increase the center back length
F3. Dipping in at the front below the waist	The skirt is not anchored at the waist because of a lack of 3D shaping	Add waist darts

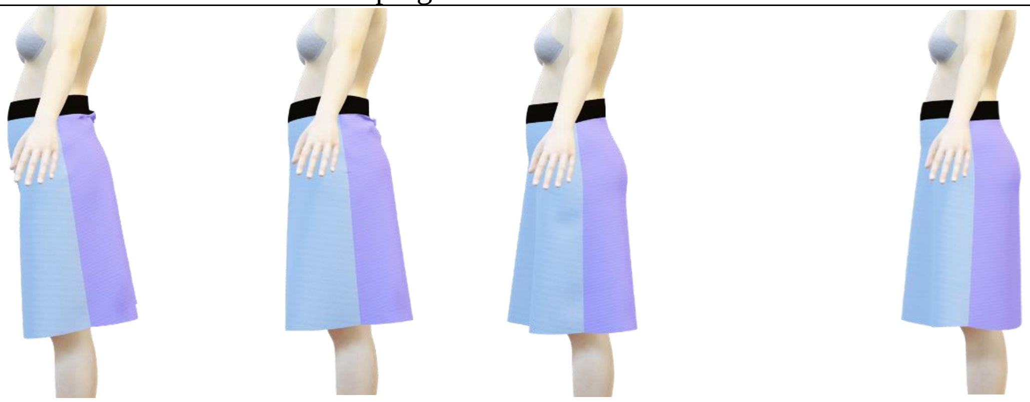

F1 F2 F3 S
