# Supplementary material for: Design and evaluation of a problem-based learning VR module for apparel fit correction training
Source: PLoS One. 2025 Jan 9;20(1):e0311587. doi: 10.1371/journal.pone.0311587 (PMC11717263; doi:10.1371/journal.pone.0311587)
Supplement: Attachment — Submitted filename: Response to reviewers.docx [file pone.0311587.s002.docx]

Reviewers' comments:

Reviewer's Responses to Questions

**Comments to the Author**

1. If the authors have adequately addressed your comments raised in a previous round of review and you feel that this manuscript is now acceptable for publication, you may indicate that here to bypass the “Comments to the Author” section, enter your conflict of interest statement in the “Confidential to Editor” section, and submit your "Accept" recommendation.

Reviewer #3: All comments have been addressed

Reviewer #4: All comments have been addressed

Reviewer #5: (No Response)

Reviewer #6: (No Response)

Reviewer #7: (No Response)

Reviewer #8: All comments have been addressed

Thank you.

2. Is the manuscript technically sound, and do the data support the conclusions?

The manuscript must describe a technically sound piece of scientific research with data that supports the conclusions. Experiments must have been conducted rigorously, with appropriate controls, replication, and sample sizes. The conclusions must be drawn appropriately based on the data presented.

Reviewer #3: Partly

Reviewer #4: Yes

Reviewer #5: Yes

Reviewer #6: Yes

Reviewer #7: Yes

Reviewer #8: Yes

Thank you.

3. Has the statistical analysis been performed appropriately and rigorously?

Reviewer #3: Yes

Reviewer #4: Yes

Reviewer #5: Yes

Reviewer #6: Yes

Reviewer #7: Yes

Reviewer #8: Yes

Thank you.

4. Have the authors made all data underlying the findings in their manuscript fully available?

The [PLOS Data policy](https://nam12.safelinks.protection.outlook.com/?url=http%3A%2F%2Fwww.plosone.org%2Fstatic%2Fpolicies.action%23sharing&data=05%7C02%7Cbaytar%40cornell.edu%7C1ca5b602014e4045f1f608dca06b3650%7C5d7e43661b9b45cf8e79b14b27df46e1%7C0%7C0%7C638561629546537767%7CUnknown%7CTWFpbGZsb3d8eyJWIjoiMC4wLjAwMDAiLCJQIjoiV2luMzIiLCJBTiI6Ik1haWwiLCJXVCI6Mn0%3D%7C0%7C%7C%7C&sdata=8%2FuPqCBJNwR6vtC3Ad89qzZigMa4lkN54bTIZeamdQw%3D&reserved=0) requires authors to make all data underlying the findings described in their manuscript fully available without restriction, with rare exception (please refer to the Data Availability Statement in the manuscript PDF file). The data should be provided as part of the manuscript or its supporting information, or deposited to a public repository. For example, in addition to summary statistics, the data points behind means, medians and variance measures should be available. If there are restrictions on publicly sharing data—e.g. participant privacy or use of data from a third party—those must be specified.

Reviewer #3: Yes

Reviewer #4: No

All data, code, and log files are available from the Cornell eCommons data archive. The files can be accessed by following this DOI link: Data from: Design and evaluation of a problem-based learning VR module for apparel fit correction training. [dataset] Cornell University eCommons Repository. <https://doi.org/10.7298/8wsv-0q41>

Reviewer #5: Yes

Reviewer #6: Yes

Reviewer #7: Yes

Reviewer #8: Yes

Thank you.

5. Is the manuscript presented in an intelligible fashion and written in standard English?

PLOS ONE does not copyedit accepted manuscripts, so the language in submitted articles must be clear, correct, and unambiguous. Any typographical or grammatical errors should be corrected at revision, so please note any specific errors here.

Reviewer #3: Yes

Reviewer #4: Yes

Reviewer #5: Yes

Reviewer #6: Yes

Reviewer #7: Yes

Reviewer #8: Yes

Thank you.

6. Review Comments to the Author

Please use the space provided to explain your answers to the questions above. You may also include additional comments for the author, including concerns about dual publication, research ethics, or publication ethics. (Please upload your review as an attachment if it exceeds 20,000 characters)

Reviewer #3: An updated manuscript addressing previous comments and suggestions was evaluated positively. The updated submission demonstrates significant improvement and provides valuable insights relevant to the research community.

Thank you.

Reviewer #4: The authors have made all revisions in the new version. This manuscript is well-written and easy to read.

Thank you.

Reviewer #5: This paper proposed to develop a very interesting problem-based learning model for apparel fit correction using VR technology. The presented research certainly facilitates the promotion of learning outcomes of garment pattern design course. However, after reading the paper, one point still needs the author’s attention: over 70% of references are from five years ago. It is suspected that the authors are really aware of all the most recent literatures related to this topic.

Thank you. We went through the manuscript and updated 13 relevant references with the most recent literature. We highlighted them in the references. We kept the rest the way they were because they justified our methods and informed our discussions and implications of our findings.

Reviewer #6: This is an interesting paper that illustrates approaches that can be developed to better understand fit and fit issues.
Why is "apparel fit" not among the papers keywords?

Thank you, we added “Apparel fit” to the keywords.

Reviewer #7: The article deals with the design and evaluation of a problem-based VR learning module for garment fit correction training. The article is well conceptualised and gives an insight into the nature of problem-based learning and shows potential in the field of digital fashion design.
The article has been thoroughly corrected and amended. I have no additional comments.

Thank you.

Reviewer #8: By utilizing virtual reality technology, this paper proposed an interactive problem-based learning approach to help the students in understanding garment design. They ask the student to apply 3D garment design software such as CLO3D to realize 3D garment fit in a problem-based learning manner. Experimental results show that such approach can improve the spatial visualization and fit correction skills. In the revised version, the authors responded well to the previous round of review comments.
If the author can explain why these problem issues were designed and why they are effective, it can make the conclusions of the paper more feasible.

Thank you. In the Literature review, between lines 52-60, we introduced our rationale for why Problem-Based Learning is important and effective as follows: “VR provides a fun, interesting, and immersive way of learning [13]. Integration of garment simulations in a PBL module can allow higher interactivity as well as control over the animations. PBL provides learners with a task that closely resembles a real-world problem and allows them to learn by doing. Several studies have been performed on the effectiveness of PBL, but they are frequently related to Science, Technology, Engineering, and Math (STEM) education. The PBL education in the apparel design domain remains to be explored. Using multi-media technologies to develop PBL tasks has been identified as a solution to impart critical and analytical thinking to students [14]. VR can provide a platform to examine and modify prototypes in a real-world setting [15].”

We also added the following summary in the Conclusions section based on your suggestion:

Lines 512-514: “The PBL questions were designed based on relevance to real-world garment design challenges and the need to develop spatial visualization and fit correction skills required for using 3D CAD software programs.”

Lines 524-526: “ The-VR based PBL approach proved effective because it ensured engagement, provided immediate feedback and enabled quick iteration.”

[NOTE: If reviewer comments were submitted as an attachment file, they will be attached to this email and accessible via the submission site. Please log into your account, locate the manuscript record, and check for the action link "View Attachments". If this link does not appear, there are no attachment files.]

While revising your submission, please upload your figure files to the Preflight Analysis and Conversion Engine (PACE) digital diagnostic tool, [https://pacev2.apexcovantage.com/](https://nam12.safelinks.protection.outlook.com/?url=https%3A%2F%2Fpacev2.apexcovantage.com%2F&data=05%7C02%7Cbaytar%40cornell.edu%7C1ca5b602014e4045f1f608dca06b3650%7C5d7e43661b9b45cf8e79b14b27df46e1%7C0%7C0%7C638561629546557420%7CUnknown%7CTWFpbGZsb3d8eyJWIjoiMC4wLjAwMDAiLCJQIjoiV2luMzIiLCJBTiI6Ik1haWwiLCJXVCI6Mn0%3D%7C0%7C%7C%7C&sdata=yDagJB73LbmtUP4%2FBL72u1FbrbcBjWFJ08xl97wDX3Y%3D&reserved=0). PACE helps ensure that figures meet PLOS requirements. To use PACE, you must first register as a user. Registration is free. Then, login and navigate to the UPLOAD tab, where you will find detailed instructions on how to use the tool. If you encounter any issues or have any questions when using PACE, please email PLOS at [figures@plos.org](mailto:figures@plos.org). Please note that Supporting Information files do not need this step.
